# Study on the Performance of Asphalt Modified with Bio-Oil, SBS and the Crumb Rubber Particle Size Ratio

**DOI:** 10.3390/polym16131929

**Published:** 2024-07-06

**Authors:** Fengqi Guo, Zhaolong Shen, Liqiang Jiang, Qiuliang Long, Yujie Yu

**Affiliations:** 1 School of Civil Engineering, Central South University, Changsha 410075, China; fengqiguo@csu.edu.cn (F.G.); 224812246@csu.edu.cn (Z.S.); jianglq2019@csu.edu.cn (L.J.); longqiuliang@csu.edu.cn (Q.L.); 2 National Engineering Research Center of High-Speed Railway Construction Technology, Central South University, Changsha 410083, China

**Keywords:** SBS/CR-modified asphalt, composite crumb rubber particle size, bio-oil, aging resistance, rheological properties, micromechanism

## Abstract

To enhance the properties of SBS and crumb rubber-modified asphalts, four different amounts (5%, 10%, 15%, and 20%) of castor oil were added to crumb rubber-modified asphalts to mitigate the adverse effects of high levels of fine crumb rubber particles on the aging resistance of SBS and crumb rubber-modified asphalt. Initially, a conventional test was conducted to assess the preliminary effects of bio-oil on the high-temperature and anti-aging properties of SBS and crumb rubber-modified asphalt. Subsequently, dynamic shear rheometer and bending beam rheometer tests were employed to evaluate the impact of bio-oil on the high- and low-temperature and anti-fatigue properties of SBS and crumb rubber-modified asphalt. Finally, fluorescence microscopy and Fourier transform infrared spectroscopy were used to examine the micro-dispersion state of the modifier and functional groups in bio-oil, SBS and crumb rubber composite-modified asphalts. The experimental results indicated that bio-oil increased the penetration of SBS and crumb rubber-modified asphalt, decreased the softening point and viscosity, and significantly improved its aging resistance. The addition of bio-oil enhanced the anti-fatigue properties of SBS and crumb rubber-modified asphalt. The optimal amount of added bio-oil was identified. Bio-oil also positively influenced the low-temperature properties of SBS and crumb rubber-modified asphalt. Although the addition of bio-oil had some adverse effects on the asphalt’s high-temperature properties, the asphalt mixture modified with bio-oil, SBS, and crumb rubber still exhibited superior high-temperature properties compared to unmodified asphalt. Furthermore, fluorescence microscopy and Fourier transform infrared spectroscopy results demonstrated that bio-oil can be uniformly dispersed in asphalt, forming a more uniform cross-linked structure and thereby enhancing the aging resistance of SBS and crumb rubber-modified asphalt. The modification process involved the physical blending of bio-oil, SBS, and crumb rubber within the asphalt. Comprehensive research confirmed that the addition of bio-oil has a significant and positive role in enhancing the properties of SBS and crumb rubber-modified asphalt with different composite crumb rubber particle size ratios.

## 1. Introduction

In recent years, the continuous development of the transportation sector and increasing traffic volume have generated a growing demand for higher quality and better performance in road construction [1,2]. Asphalt material, as a fundamental component of road construction, has an essential role in the lifespan and load-bearing capacity of roads. Due to the limitations of unmodified asphalt, polymer-modified asphalt has been proposed to realize superior performance [3,4]. Among these, styrene-butadiene-styrene (SBS) is an excellent modifier that significantly improves the ductility and viscoelasticity of asphalt [5], thereby greatly enhancing its high- and low-temperature and anti-aging properties. However, when the SBS content exceeds 5% in practical road construction, clustering and agglomeration phenomena easily occur in the asphalt [6], which adversely affects the modification effect [7]. The modifier content of SBS-modified asphalt (SBSMA) is usually 5%-8%. When the SBS content is further increased, the storage stability of asphalt will significantly decrease [8], and the economic feasibility will also decrease significantly [9].

To address the limitations of SBSMA, many researchers have proposed SBS/CR composite-modified asphalt (SCMA) [10,11,12], which incorporates crumb rubber (CR) to replace a portion of SBS. The CR is derived from waste tires at low cost [13,14]. The adoption of CR not only facilitates waste recycling but also reduces the construction cost of SBSMA. Additionally, CR significantly enhances the high-temperature properties, anti-aging properties, and durability of modified asphalt [15]. Some researchers have suggested a high dosage of CR to achieve a modification effect comparable to that of SBS-modified asphalt [16]. However, a high dose of CR will lead to excessive viscosity and poor compatibility with SCMA. The storage stability of the modified asphalt will also be compromised [17]. Zhang et al. [18] revealed that the combination of CR with SBS increased the viscosity of the modified asphalt and improved its sensitivity to temperature. However, as the mixing temperature increased, the viscosity decreased, and the temperature sensitivity decreased, which was attributable to the degradation of SBS.

Moreover, the fineness of the CR particles influences the modification effect differently. In a previous study [19], CRMA with different composite CR ratios of 80 mesh, 60 mesh, and 40 mesh exhibited superior high- and low-temperature properties. The asphalt was compatible with the modifier, and the modified asphalt presented increasing properties with a greater percentage of fine particles (80 mesh). However, as aging progresses, the fine CR particles gradually degrade. Therefore, it is essential to find a solution to mitigate the impact of fine CR particle degradation on SCMA performance. It is also important to address the issues of excessive viscosity due to the high CR dosage and poor compatibility between the modifier and asphalt to ensure the construction and workability of SCMA.

Bio-oil, sourced from various biomass pyrolysis products [20] and byproducts of the petroleum industry [21,22], exhibits several advantageous properties. It is abundant, inexpensive, and renewable. Bio-oil is rich in light-weight components and has excellent compatibility with asphalt [23,24]. These attributes make bio-oil a viable strategy for addressing the stability, compatibility, and aging deficiencies in modified asphalt [25]. Ma et al. [26] reported that the addition of waste cooking oil to CRMA promoted the expansion and dissolution of CR particles, which improved the asphalt storage stability and decreased the viscosity of CRMA. Lyu et al. [27] reported that the inclusion of bio-oil in CRMA increased the resistance to viscous damage threefold. Bio-oil-modified rubber (BMR) exhibited superior aging resistance. Huang et al. [28] pretreated CR with bio-oil and found that the high-temperature properties of SCMA decreased. However, the storage stability of the modified asphalt improved. Wen et al. [29] reported that the fatigue life of SBSMA was significantly reduced by long-term aging. However, by adding bio-oil to aged modified asphalt, the fatigue resistance of the modified asphalt was restored. Nizamuddin et al. [30] demonstrated that bio-oil extracted from waste plastics, when used as a rehabilitating agent at 5% and 8% contents, provided functional components to asphalt and enhanced the aging resistance. Lei et al. [31] prepared BMR using corn stover bio-oil at contents ranging from 5% to 15%, along with 20% CR. The results indicated that bio-oil promoted the further expansion of CR during aging, enhancing the elasticity and aging resistance of the asphalt. Zhou et al. [32] researched the stability and rheological properties of different types of BMR and sulfur incorporating 15% bio-oil and 15% CR.

The study showed that castor oil and vegetable oil effectively improved the interaction between asphalt and sulfur, thereby enhancing the viscoelasticity of asphalt. Yang et al. [33] investigated the rheological characteristics of composite-modified asphalt containing 1.5% polyphosphoric acid, 2.5% SBS, and unmodified asphalt to create bio-asphalt (85:15). The results indicated that bio-oil reduced the high-temperature properties of SBS-modified asphalt but also increased its low-temperature and anti-aging properties.

In summary, bio-oil not only enhances the low-temperature, anti-aging, and anti-fatigue properties of modified asphalt but also increases the utilization of CR while ensuring the construction and workability of the modified asphalt. Therefore, the core objective of this study was to investigate the influence of adding bio-oil on the performance of SCMA composites with different CRs. In this study, castor oil was selected for SCMA modification. Initially, the effects of bio-oil addition on the high-temperature and anti-aging properties of SCMA were preliminarily analyzed through penetration and softening point tests. Subsequently, the viscosity of the modified asphalt was determined by the Brookfield viscosity test, and its aging resistance was evaluated. Based on the DSR and BBR tests, the effects of bio-oil addition on the asphalt’s high- and low-temperature properties, together with the fatigue resistance of SCMA before and after aging, were further investigated. Additionally, FM and FTIR tests were conducted to analyze the microdispersion state of the modifier and to investigate the functional component variations in bio-oil-modified asphalt (B-SCMA). These analyses aimed to elucidate the micromechanism of the modification and provide both theoretical and practical support for improving the performance of the SCMA.

## 2. Materials and Methods

### 2.1. Materials

Panjin 90# road petroleum asphalt was used as the unmodified asphalt (UA), and its main technical indicators were verified to follow the JTG E20-2011 specification. Table 1 shows the main technical indicators of the UA. The SBS modifier was provided by Beijing Guolu High-tech Company (Beijing, China), and its relevant technical indicators are detailed in Table 2. The CR was supplied by Guangxi Jiaoke Company (Guangxi, China), and Table 3 shows its main technical specifications. The bio-oil was castor oil that had been extracted from the distillation residue of castor seeds, and its relevant characteristics are outlined in Table 4. The castor oil used is produced by Nantong Yuhao Chemical Technology Company (Nantong, China).

### 2.2. Test Method

#### 2.2.1. Preparation of the Modified Asphalt

Working according to previous investigations [25], the UA was first heated to a flowable state at 135 °C. Then, the SBS modifier (2%) was added, and the sample was sheared for 5 min at 4500 rpm and 170–180 °C. After that, 15% of the composite CR with a specific particle size ratio was incorporated. Based on preliminary research [19], a ratio of 6:2:2 (80 mesh, 60 mesh, and 40 mesh) with a greater proportion of fine CR particles was selected. The mixture was then sheared for 30 min at 4500 rpm and 170–180 °C. Finally, the prepared SCMA was cured in an oven at 170 °C for 60 min.

**Figure 1:**
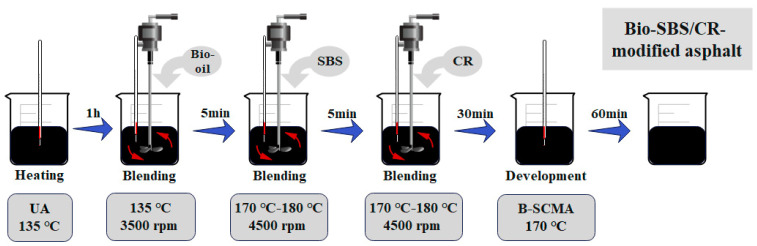
Preparation process of B-SCMA.

For B-SCMA, the UA was heated to a flowable state at 135 °C, and then 5%, 10%, 15%, and 20% castor oil were added. Subsequently, the mixture was sheared for 5 min at 3500 rpm and 135 °C. Next, 2% of the SBS modifier was added to the bio-oil-modified asphalt, and the mixture was sheared for 5 min at 4500 rpm and 170–180 °C. Next, 15% of the composite CR with a particle size ratio of 6:2:2 was incorporated, and the mixture was sheared for 30 min at 4500 rpm and 170–180 °C. Finally, the prepared B-SCMA was cured in an oven at 170 °C for 60 min. Figure 1 illustrates the preparation process of B-SCMA, and Table 5 provides the abbreviations used for modified asphalt under different modifier dosages and preparation conditions.

#### 2.2.2. Conventional Performance Tests

Working according to the AASHTO D 1754 [34] and AASHTO D 6521 [35] standards, short-term and long-term aging tests were performed on unmodified asphalt and various modified asphalts using a rolling thin film oven (RTFO) and a pressure aging vessel (PAV), respectively. The RTFO test involved aging the asphalt in aging bottles at 165 °C for 85 min. The short-term-aged asphalt was subjected to a pressure of 2.1 MPa and a temperature of 100 °C for 20 h.

Penetration, softening point, and Brookfield viscosity tests were conducted according to ASTM D 5 [36], ASTM D 3461 [37], and AASHTO T 316, respectively. The penetration ratio and softening point change rate of various asphalts were calculated using Equations (1) and (2). Equation (3) was employed to calculate the aging index of various asphalts, based on the Brookfield viscosity test results, thereby further evaluating the aging resistance of B-SCMA:

(1) $$PR=\frac{P_{Original}}{P_{RTFO}}$$

(2) $$SPR=\frac{{SP}_{RTFO}-{SP}_{Original}}{SP_{Original}}$$

(3) $$CI=lglg\left(\eta_{RTFO}\times 10^{3}\right)-lglg\left(\eta_{Original}\times 10^{3}\right)$$

where PR is the penetration ratio. $P_{Original}$ is the penetration value before aging. $P_{RTFO}$ is the penetration value after aging. SPR is the softening point change rate. ${SP}_{Original}$ is the softening point before aging. ${SP}_{RTFO}$ is the softening point value after aging. CI is the aging index of the asphalt. $lglg\left(\eta_{Original}\right)$ is the viscosity before aging. $lglg\left(\eta_{RTFO}\right)$ is the viscosity after aging.

#### 2.2.3. Dynamic Shear Rheometer (DSR) Test

Working according to the standard AASHTO T 350 [38], the DSR test was conducted using an Anton Paar MCR 302e (Anton Paar, Shanghai, China) device. The temperature sweep (TS) test was performed at a strain of γ = 12%, a frequency of ω = 10 rad/s, and temperatures ranging from 46 to 88 °C. The complex shear modulus (G*) was measured to evaluate the asphalt’s high-temperature properties. The frequency sweep (FS) test was used to evaluate the complex modulus (G*), phase angle (*δ*), and rutting index (G*/sin δ) at different frequencies at 46 °C and was used to evaluate the deformation resistance of various asphalts under varying frequency loads. Equation (4) was employed to analyze the temperature sensitivity of various asphalts:

(4) $$\mathrm{Lg}\;G^{*}/sin\;\delta =aT+b$$

where T is the temperature (°C). $G^{*}/sin\;\delta$ is the asphalt rutting index (kPa). a is the slope of the fitted curve. b is the intercept of the fitted curve. $R^{2}$ is the fitting correlation coefficient.

LAS testing was conducted on long-term-aged asphalt to evaluate the fatigue performance. The tests were divided into frequency sweeps and amplitude sweeps. Frequency sweep tests were conducted at 25 °C, with a strain amplitude of 0.1% and a frequency range of 0.2 to 30 Hz. The linear amplitude sweep used strain control mode, with a scanning duration of 300 s. The frequency was 10 Hz, and the strain range was increased from 0.1% to 30%. The fatigue life was calculated using Equation (5), with parameters derived from the LAS test:

(5) $$N_{f}={A(\gamma_{max})}^{B}$$

where $N_{f}$ is the asphalt fatigue life. $\gamma_{max}$ is the peak strain (%). A and B are important parameters in the fatigue life equation.

#### 2.2.4. Bending Beam Rheometer Test (BBR)

Working according to the standard T 313 [39], the BBR test was conducted using TE-BBR equipment (Cannon, Guangzhou, China). This test assessed the low-temperature properties of the asphalts by evaluating their stiffness modulus and creep rate at −12 °C, −18 °C, and −24 °C. The values for S and m were measured at 60.0 s under a load of 980 ± 50 mN.

#### 2.2.5. Fluorescence Microscopy (FM)

FM is a common method for observing the microstructure of polymer-modified asphalt. In this study, the fluorescent substances introduced by SBS and CR in the modified asphalt were observed to analyze the microdispersion of the materials before and after the addition of bio-oil. The impact of aging on the microdispersion of SBS and CR in B-SCMA was compared between unaged and PAV-aged B-SCMA. Leica DFC 7000T (Leica, Shanghai, China) equipment at 200× magnification was used for the FM test.

#### 2.2.6. Fourier Transform Infrared (FTIR) Spectroscopy

FTIR is the primary method for analyzing functional groups in polymer-modified asphalt. In this study, FTIR was used to observe changes in the absorption peaks of the infrared spectrum. This analysis aimed to investigate the composition and alterations of functional groups in B-SCMA after the addition of bio-oil and to determine possible chemical reactions. A Thermo Fisher IS50 (Thermo Fisher, Shanghai, China) instrument was used for FTIR spectroscopy, with a resolution of 4 cm^−1^ and 32 scans per sample, covering a wavenumber range of 4000 cm^−1^ to 500 cm^−1^.

## 3. Results and Discussion

### 3.1. Conventional Performance Tests

**Figure 2:**
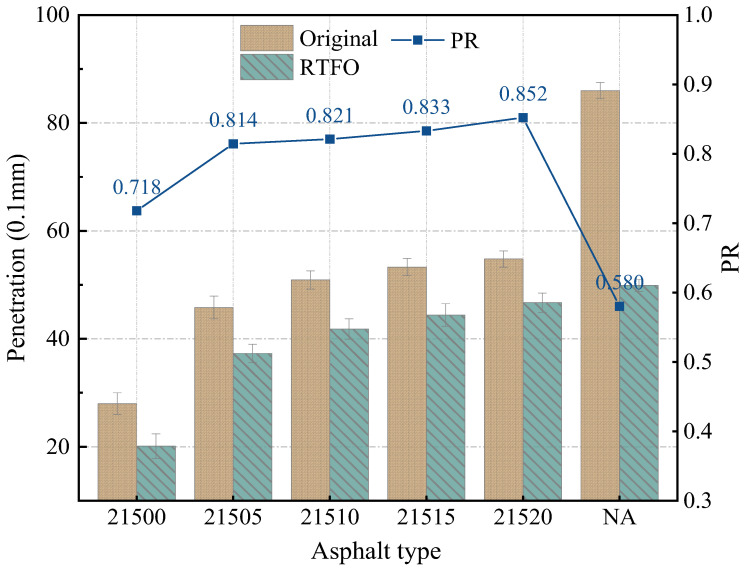
Penetration test results.

**Figure 3:**
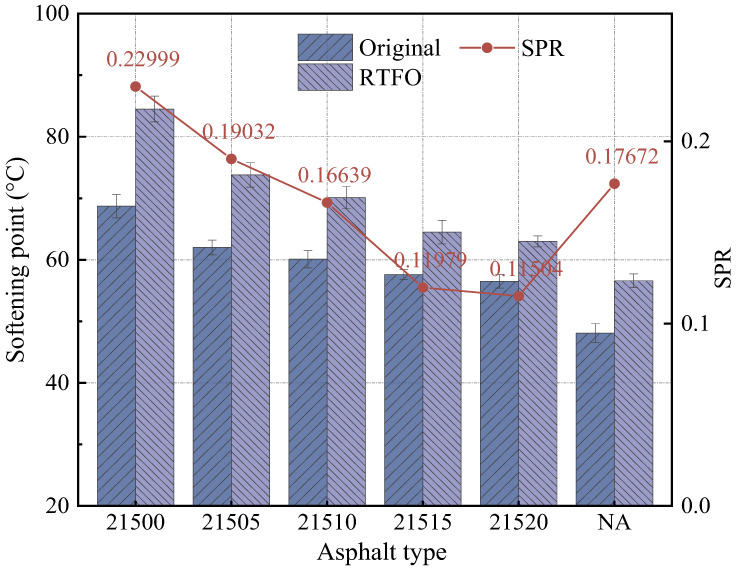
Softening point test results.

The influence of adding bio-oil on the conventional performance of SCMA with different CR particle size ratios was initially evaluated by measuring the penetration and softening points before and after treatment in the RTFO. Subsequently, using Equations (1) and (2), the variation rates of the penetration ratio and softening point were measured to evaluate the aging resistance of B-SCMA under different CR particle size ratios. The test results are shown in Figure 2 and Figure 3.

According to Figure 2, the penetration of UA was the highest both before and after treatment in the RTFO. With the addition of SBS, CR, and bio-oil, the penetration of the modified asphalts significantly decreased. The addition of modifiers improved the internal structure of the asphalt, making the modified asphalt harder. Notably, the penetration value was the lowest at 21500 when only SBS and CR were added. However, as the percentage of bio-oil increased, the penetration of the modified asphalts tended to increase. This is because adding bio-oil replenishes the light-weight components in the SCMA, making the SCMA softer and thereby increasing its penetration value. After RTFO treatment, the penetration values of all asphalts decreased, due to the volatilization of light-weight components.

However, the penetration changing rates before and after aging differed among the various asphalts, indicating different anti-aging performances. The PR test is used for evaluating the aging resistance of asphalt by analyzing the changes in hardness during the aging process. A smaller penetration ratio implies harder asphalt after passing through the RTFO, which also indicates poorer aging resistance. The order of the PR values for the six asphalts was UA < 21500 < 21505 < 21510 < 21515 < 21520 (Figure 2). The PR value of 21520 was 18.7% greater than that of 21500. Clearly, bio-oil plays a positive role in improving the anti-aging properties of SCMA.

The softening point is an important indicator for assessing the high-temperature properties of asphalt. The softening point of UA was the lowest both before and after RTFO treatment, while the softening point of the sample without bio-oil was the highest. The softening point of the modified asphalt gradually decreased as the bio-oil content increased. This reduction can be attributed to the light-weight components in the bio-oil. When bio-oil is added to asphalt, the replenished light-weight components decrease the proportion of asphaltene, thereby decreasing the high-temperature properties of the asphalt. The softening point of 21520 is 56.5 °C, which is still 8.4 °C higher than that of UA, indicating that B-SCMA retains better high-temperature performance than UA.

After RTFO treatment, the softening points increased to different degrees, which indicates that the modified asphalts exhibited different aging resistances. The softening point change rate (SPR) evaluates the variation in the softening point before and after RTFO treatment and serves as an indicator of the anti-aging properties. A lower SPR indicates better anti-aging performance. As shown in Figure 3, the SPR gradually decreased with increasing bio-oil content. Specifically, the SPR of 21520 was 49.9% lower than that of 21500, which indicates that adding bio-oil significantly improved the anti-aging properties of SCMA. Bio-oil mitigates the loss of light-weight components and fine CR particles during aging, thereby replenishing the lost light-weight components and enhancing the anti-aging performance of SCMA.

### 3.2. Viscosity Test

**Figure 4:**
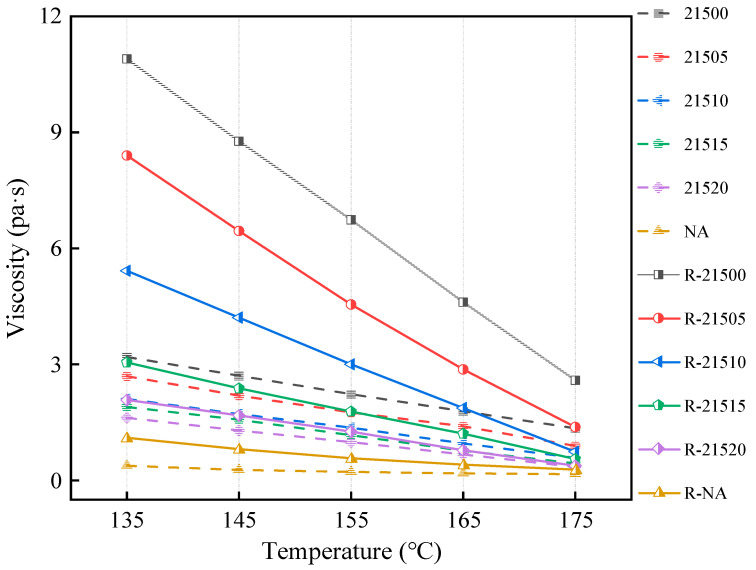
Viscosity test results.

**Figure 5:**
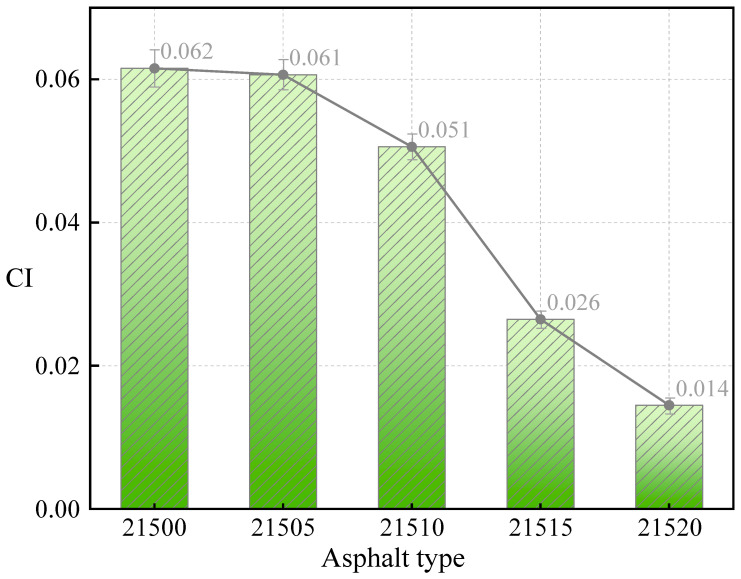
Viscosity changes of modified asphalt.

The viscosity reflects the asphalt’s macroscopic response to changes in the relative molecular weight. Since aging inevitably affects viscosity, the viscosity changes before and after aging are crucial indicators of the asphalt aging process. Brookfield viscosity tests were conducted to evaluate the influence of bio-oil on the viscosity of SCMA with composite CR particle size ratios, and the results are shown in Figure 4. The changes in viscosity before and after RFTO treatment were compared to evaluate the anti-aging properties, as shown in Figure 5.

The viscosity decreased with increasing temperature, both before and after RTFO treatment. This phenomenon occurs because higher temperatures enhance the thermal movement of asphalt molecules, thereby reducing the overall flow resistance and viscosity. Before and after RTFO treatment, the asphalt viscosity decreased in the order of UA < 21520 < 21515 < 21510 < 21505 < 21500. At 135 °C, the viscosity of 21500 was 740% greater than that of UA. After RTFO treatment, the viscosity of R-21500 increased significantly, negatively impacting the workability. However, the viscosity of SCMA decreased to different degrees with the addition of bio-oil. This also indicates that the addition of bio-oil has a positive effect on the construction workability of the modified asphalt. Specifically, at 135 °C, the viscosity of 21520 was 49.2% lower than that of 21500. As previously discussed, the addition of bio-oil replenishes the light-weight components in asphalt, reduces the proportion of asphaltenes, and results in a softer and more fluid asphalt.

The increase in viscosity before and after RTFO treatment varies among different asphalts, reflecting differences in their anti-aging performance. The aging index (CI) at 135 °C, calculated using Equation (3), characterizes the anti-aging performance of asphalt. A smaller CI indicates better anti-aging performance. As shown in Figure 5, the CI decreased with the addition of bio-oil. This decrease occurred because bio-oil mitigates the degradation of fine CR particles in SCMA due to aging, thereby enhancing the aging resistance. Although the difference in CI between 21505 and 21500 was small, the CI decreased more significantly as the bio-oil content increased. Specifically, the CI of 21520, which had the highest bio-oil content, was 76.5% lower than that of 21500. The CI value of 21515 is 56.9% lower than that of 21500. Overall, the bio-oil significantly improved the aging resistance of the SCMA, reduced the excessive viscosity caused by the high CR content, thereby improving the workability of the modified asphalt.

### 3.3. Rheological Tests

#### 3.3.1. Temperature Sweep Test

**Figure 6:**
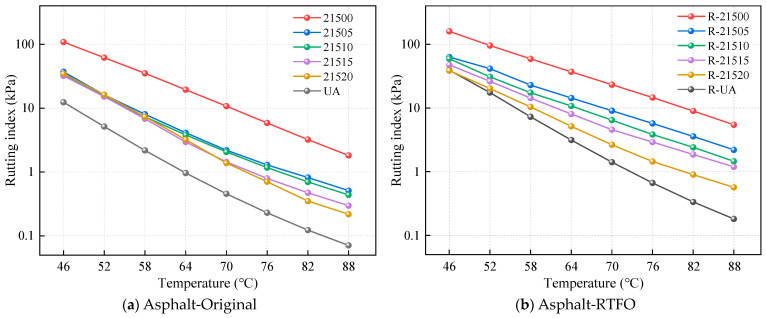
Rutting index test results.

To evaluate the effects of bio-oil on the high-temperature performance of SCMA composites with different CR particle size ratios, this section examines the rutting indices of unmodified asphalt and various modified asphalts before and after RTFO treatment through TS tests. The rutting index is a critical indicator of high-temperature performance, and high index values indicate better resistance to high-temperature deformation. The rutting index test results before and after RTFO treatment are shown in Figure 6.

Since asphalt becomes more fluid at higher temperatures, the rutting indices of various asphalts decrease with increasing temperature in the unaged state, as shown in Figure 6a. The chemical properties of bio-oil and its compatibility with asphalt influence its melting characteristics and high-temperature properties, reducing its resistance to deformation under external forces. Notably, UA exhibited the lowest rutting index value, while 21500 displayed the highest value. At 76 °C, the rutting index of 21500 was 2450% greater than that of UA, indicating that SCMA with a high composite CR particle size ratio had excellent high-temperature properties. This is attributed to the creation of a denser microspatial structure in SCMA due to the composite CR particles, which enhances its high-temperature performance [19]. Additionally, the rutting index of B-SCMA gradually decreased with increasing bio-oil content, indicating that bio-oil negatively impacts the high-temperature performance of modified asphalt. The addition of bio-oil changes the asphalt component ratios. An increase in the amount of light-weight components used reduces the high-temperature properties of modified asphalt [40]. However, B-SCMA with various bio-oil contents still demonstrated superior high-temperature performance compared to that of UA. The variation in the rutting index of B-SCMA with temperature was less pronounced at 46–64 °C than at higher temperatures. The light-weight components in bio-oil are easily lost at elevated temperatures, contributing to the temperature sensitivity of B-SCMA.

Figure 6b illustrates the change in the rutting index after RTFO treatment at different temperatures. The order of the rutting indices of several modified asphalts is more pronounced between 46 and 64 °C than before short-term aging. The rutting index of asphalt increased significantly after aging compared to that in the unaged state. The loss of light-weight components and an increase in asphaltene content induced hardening of the asphalt. However, the degree of increase in the rutting index varied among the different asphalts. The change in the rutting index of modified asphalts before and after RTFO treatment was less pronounced with the addition of bio-oil, indicating enhanced anti-aging performance. This improvement can be attributed to the increase in aromatic content in the asphalt with bio-oil addition [41]. A higher aromatic content can replenish volatile light-weight components lost during aging, thereby enhancing the anti-aging performance [42].

The varying slopes of the rutting index indicate distinct temperature sensitivities. To quantitatively evaluate the temperature sensitivity, the rutting index for each asphalt sample was calculated using Equation (4). The test results are shown in Table 6. Adding bio-oil to unmodified asphalt can alter its temperature sensitivity [22]. In Table 6, the “a” value represents the slope of the fitted curve and the temperature sensitivity of the asphalt. A smaller “a” value indicates lower temperature sensitivity, meaning that the rutting index changes less with temperature. The “a” values for the five modified asphalts were in the following order: 21520 < 21515 < 21510 < 21505 < 21500. With increasing bio-oil content, the temperature sensitivity of the modified asphalt decreased, indicating the improved compatibility of various modifiers in B-SCMA.

#### 3.3.2. Frequency Sweep Test

**Figure 7:**
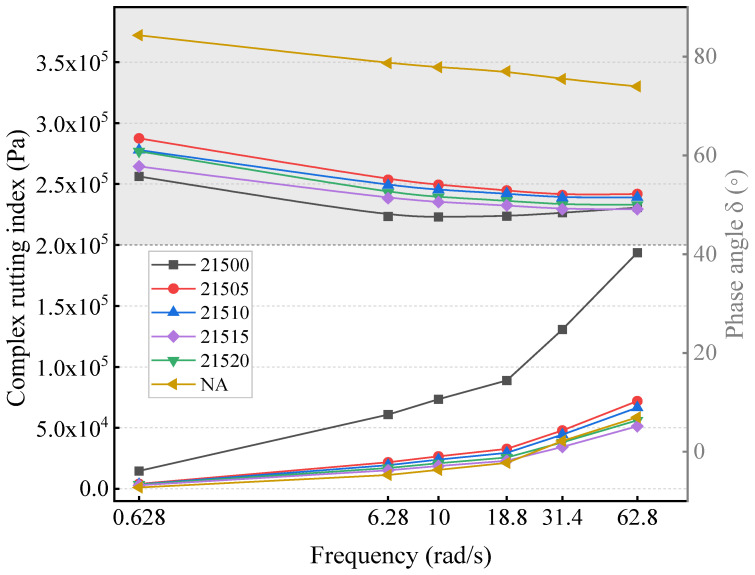
Frequency sweep test results.

The FS test is a dynamic mechanical testing method that is primarily employed to evaluate the viscoelastic characteristics of asphalt. This test involves applying dynamic loads at various frequencies to measure the G* and *δ* of the asphalt, which are then used to calculate the rutting index. This calculation helps evaluate the mechanical response under different vibration frequencies. Figure 7 shows the variation curves of G* and *δ* with frequency for the modified asphalts at 46 °C.

**Figure 8:**
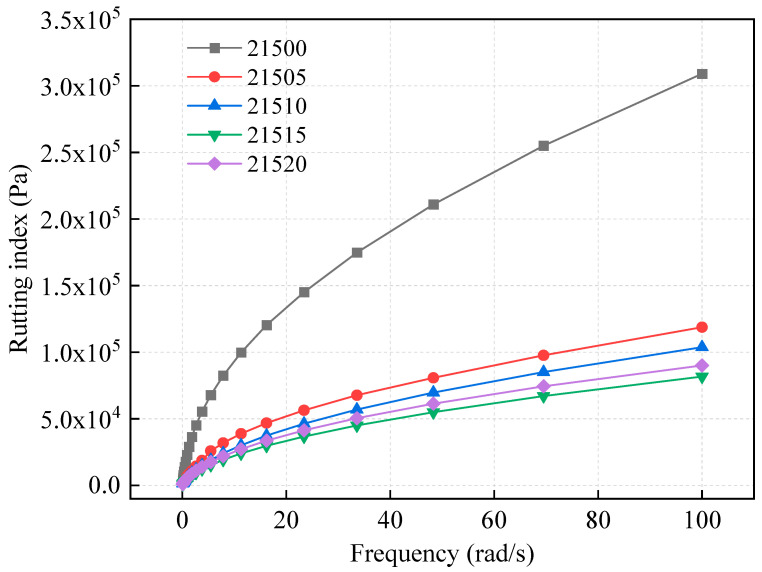
Frequency-retrieval index curves.

As depicted in Figure 7, the complex modulus increases with frequency, while the phase angle decreases [43]. At the same frequency, the deformation resistance increases with increasing complex modulus and decreasing phase angle. Notably, in our tests, UA exhibited the weakest deformation resistance, whereas 21500 demonstrated the best deformation resistance. At the same frequency, the complex shear modulus of the modified asphalts followed the order 21500 > 21505 > 21510 > 21520 > 21515, with the phase angle order being the reverse. The shear deformation resistance of the modified asphalts initially decreased with increasing bio-oil content, but this trend reversed when the bio-oil amount surpassed 15%. This indicates the optimal bio-oil content for B-SCMA. To comprehensively analyze the effect of different bio-oil contents on the deformation resistance of B-SCMA, Figure 8 compares the rutting indices of modified asphalts with frequency.

Figure 8 illustrates that at 46 °C, the rutting index of asphalt increases with frequency, indicating that the deformation resistance is greater under high-speed loading than under low-speed loading. At the same frequency, the order of rutting indices for the modified asphalts in our study was 21500 > 21505 > 21510 > 21520 > 21515. It is evident that the rutting indices of the four bio-oil-modified asphalts were significantly lower than those of the asphalts without added bio-oil. This result is consistent with the temperature scanning test results, showing that bio-oil reduced the high-temperature properties of SCMA. The rutting index of modified asphalt usually decreases as the bio-oil content increases. This decrease can be attributed to the decrease in viscosity caused by the addition of bio-oil, which diminishes the high-temperature properties and weakens the resistance to pavement deformation under loads [44].

However, the 21520-modified asphalt demonstrated superior high-temperature deformation resistance compared to 21515. This observation aligns with the temperature sweep test results, where the rutting index of unaged 21520 was greater than that of 21515 up to 70 °C. However, the rutting index of R-21515 exceeded that of 21520 at temperatures higher than 70 °C. After RTFO treatment, the rutting index of R-21515 remained higher than that of R-21520. This suggests that initially, a higher bio-oil content did not significantly affect the high-temperature performance. As temperature and aging increase, the influence of temperature on the rutting index of asphalt becomes more pronounced. This is due to the complementary or compensatory effect of bio-oil modification and the enhancement provided by SBS or CR modifiers, which temporarily mask the negative impact on high-temperature performance.

#### 3.3.3. LAS Test

**Figure 9:**
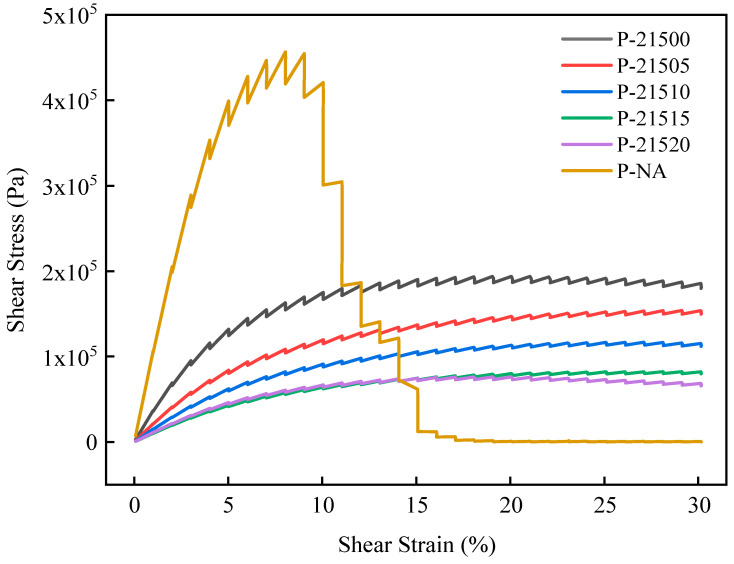
LAS test results.

**Figure 10:**
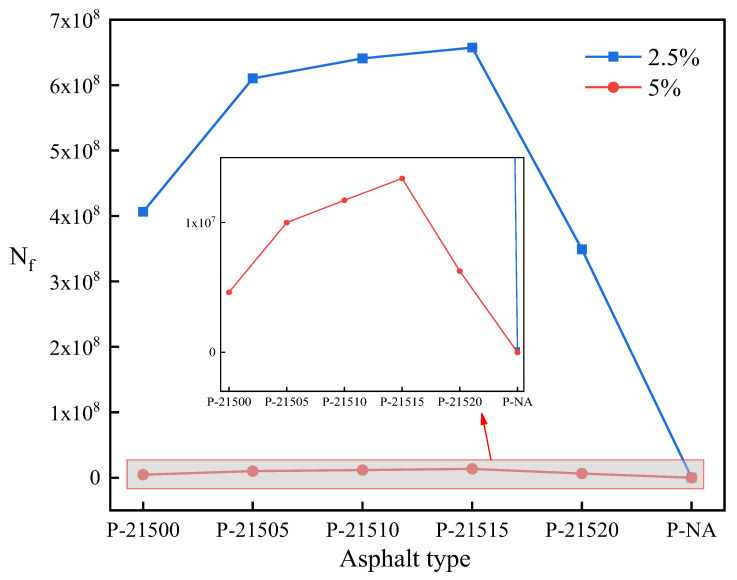
Fatigue life results.

This section investigates the impact of bio-oil on the fatigue resistance of SCMA using LAS tests. The stress-strain curves of the asphalt were derived, and the fatigue performance was quantified using Equation (5). Figure 9 presents the stress-strain curves obtained from the LAS tests, while Figure 10 illustrates the fatigue life of each asphalt type.

According to the LAS test results depicted in Figure 9, the stress-strain curve of the UA reaches its peak first, followed by a sharp decline in stress with increasing strain, leading to premature stress reduction to the lowest level. This indicates that UA exhibits the earliest fatigue failure and the poorest fatigue resistance. In contrast, the stress peaks of the other modified asphalts are notably lower than those of UA, and their stress changes with strain more gradually, suggesting superior fatigue resistance compared to that of UA. The stress-strain curve of P-21500, which contains only SBS and CR, is positioned above the curves of the five B-SCMA samples with bio-oil. This trend indicates that bio-oil addition enhanced the fatigue resistance of SCMA composites with different CR particle size ratios. This enhancement can be attributed to the ability of bio-oil to inhibit the degradation of fine CR particles during aging, thereby stabilizing the dense network structure inherent in SCMA. Notably, the positions of the P-21505 and P-21510 curves differ significantly. At low strain levels, the stress-strain curve of P-21520 is greater than that of P-21515. As the strain increases, the stress-strain curve of P-21515 surpasses that of P-21520, suggesting that P-21520 had inferior fatigue resistance compared to P-21515. This indicates that increasing the bio-oil content does not necessarily improve the fatigue resistance of modified asphalt. To better understand the anti-fatigue properties of various asphalts, the fatigue lives were calculated and are compared in Figure 10.

Figure 10 illustrates the fatigue lives of the six tested asphalts at different stress levels. It is clear that the fatigue life of asphalts subjected to 2.5% stress is significantly longer than that of those subjected to 5% stress. This disparity arises because fatigue failure is cumulative damage and is linearly correlated with the stress amplitude magnitude. Consequently, a lower stress level (e.g., 2.5%) implies that the material experiences less stress per cycle, resulting in reduced microdamage in each cycle and an extended fatigue life. As the bio-oil content increased, the fatigue life of B-SCMA initially increased before decreasing. At a stress level of 2.5%, the fatigue life of P-21515 was improved by 61.8% compared with that of P-21500. This trend aligns with previous analyses, indicating that an increase in bio-oil content does not necessarily improve the fatigue resistance of modified asphalt. An optimal amount of bio-oil may improve the fluidity of modified asphalt, promoting a more uniform micro-distribution structure. This enhancement may mitigate the local stress contents arising from uneven mixing, thereby initially extending the fatigue life. However, excessive bio-oil addition diminishes the viscosity and cohesion of asphalt, increasing the susceptibility of the material to flow and deformation under repetitive loading. This vulnerability to deformation undermines the asphalt’s capacity to resist fatigue crack propagation, ultimately reducing the fatigue life of the asphalt.

#### 3.3.4. BBR Test

**Figure 11:**
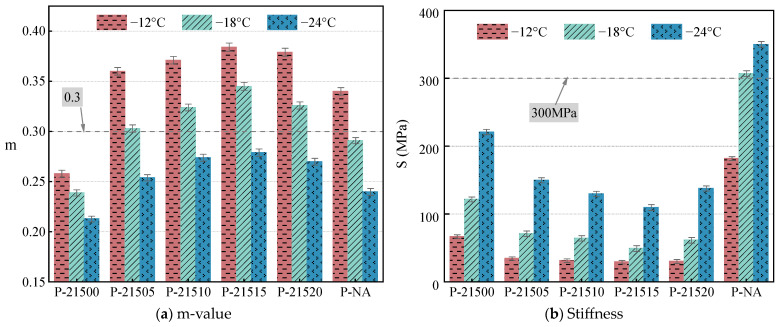
BBR test results.

To investigate the effect of bio-oil on the low-temperature properties of SCMA with diverse composite CR particle size ratios, the creep rate (m) and stiffness modulus (S) of various asphalts were assessed using the BBR test, and the results are presented in Figure 11.

Figure 11a,b depict the m and S values for both unmodified and modified asphalts. High m values and low S values indicate superior low-temperature properties. The low-temperature properties of the five modified asphalts were ranked as follows: P-21500 < P-21505 < P-21510 < P-21520 < P-21515. The lowest low-temperature properties of P-21500 correspond to the highest high-temperature properties, which also indicates that the high- and low-temperature properties of modified asphalt are usually the opposite of each other. Figure 11 reveals that as the bio-oil content increased, the m value of the modified asphalt initially increased before decreasing. The S value decreased initially but later increased, particularly when the bio-oil content surpassed 15%. The low-temperature properties of the modified asphalt with bio-oil percentages below 15% may be attributed to the stiffness reduction effect facilitated by the bio-oil. The low-temperature performance is enhanced at the expense of high-temperature performance [45]. However, when the bio-oil amount exceeds 15%, the low-temperature performance of B-SCMA deteriorates, which may be attributed to an excessive compromise with the use of bio-oil on the stability of the SCMA microstructure. This mechanism will also diminish the cohesion properties and impair the elastic recovery of asphalt at low temperatures.

### 3.4. FM Test

**Figure 12:**
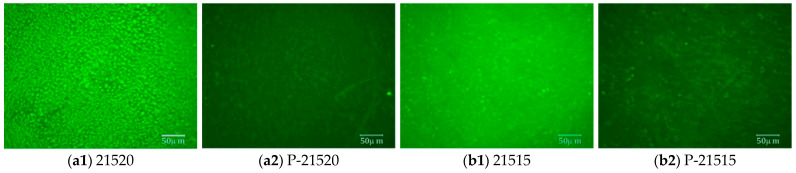

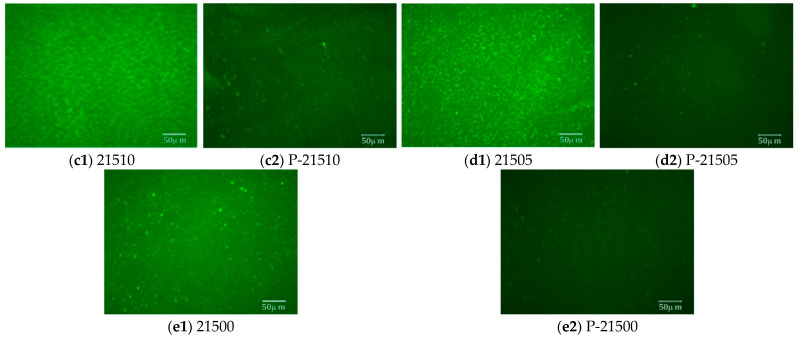
Fluorescence micrographs of B-SCMA. (**a1**)–(**e2**) correspond to different states and types of asphalt.

Fluorescence microscopy (FM) is a valuable tool for examining modifier particle distributions and for elucidating the micro interactions between modifiers and asphalt. In this study, FM was utilized to evaluate the impact of bio-oil on the distribution, uniformity, and aging resistance of SCMA. Fluorescence micrographs of B-SCMA under various bio-oil contents, both before and after PAV, were acquired and are presented in Figure 12.

As illustrated in Figure 12, the proportion of fluorescent substances in B-SCMA increased with the addition of bio-oil, accompanied by morphological changes in B-SCMA. Compared to the SCMA results shown in Figure 12(e1), the B-SCMA samples with 5% and 10% bio-oil contents (Figure 12(c1,d1)) exhibited a more uniform dispersion of fluorescent substances and an increase in light-weight components within the asphalt. This occurs because bio-oil enhances the light-weight components of asphalt, promoting the swelling and uniform dispersion of SBS and CR modifiers into punctate shapes. This indicates that bio-oil improves the compatibility between asphalt and the SBS and CR modifiers, facilitating better dispersion. Figure 12(b1) shows that with 15% bio-oil content, B-SCMA formed a more uniform cross-linked structure with filamentous features, suggesting a richer network structure due to the continuous swelling of modifiers and bio-oil. However, when the bio-oil content reached 20%, as shown in Figure 12(a1), the continuous network structure disappeared and was replaced by larger cluster structures. This implies the existence of a threshold at approximately 15% of bio-oil content, beyond which the further addition of bio-oil does not enhance the modification effect. Excessive bio-oil disrupts the original spatial network structure, causing segments of the modifiers to aggregate with bio-oil molecules, resulting in phase separation structures that appear as clusters under fluorescence microscopy [46].

Upon comparing the images of modified asphalt before and after PAV, it is evident that the fluorescence intensity decreases aging, with B-SCMA demonstrating enhanced aging resistance relative to SCMA as the bio-oil content increases. This improvement can be attributed to the esters in castor oil, which can retard or inhibit free radical chain reactions, thereby reducing asphalt oxidation and enhancing aging resistance [47]. However, an increase in bio-oil does not invariably lead to better aging resistance. As depicted in Figure 12(a2,b2,c2,d2), B-SCMA with 15% bio-oil content exhibited the most significant retention of fluorescent substances after aging. This suggests that while bio-oil initially enhances the compatibility and aging resistance of SCMA by increasing the amount of light-weight components and promoting SBS and CR swelling, an excess content of bio-oil can disrupt the stability of the SCMA spatial network structure once the swelling capacity is saturated. As a result, aging resistance is impaired. Within an optimal range of bio-oil contents, the compatibility and aging resistance of the SCMA improved. However, exceeding this threshold led to a reduction in modification efficacy due to the destabilization of the SCMA spatial network structure.

The physical blending and synergistic reaction mechanism of bio-oil plays a significant role in promoting compatibility between the SBS/CR modifier and unmodified asphalt. Bio-oil helps to reduce the interfacial tension between different components, which encourages the polymer chains of SBS and CR to be more uniformly distributed in the asphalt. The phase separation phenomenon decreases, and a more homogeneous and stable dispersion is obtained. This optimized dispersion state not only enhances the interaction between the modifier and asphalt but also effectively improves the asphalt’s performance. Furthermore, the asphalt’s anti-aging properties, mobile phase, and compatibility are improved due to the formation of a denser network structure. However, the modification effect will be reduced by an excessive dosage of bio-oil.

### 3.5. FTIR Test

**Figure 13:**
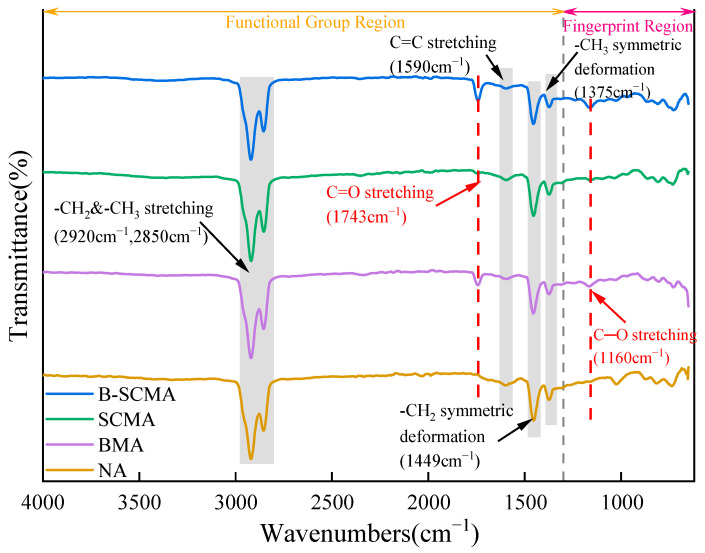
The infrared spectra of UA, SCMA, BMA, and B-SCMA.

**Figure 14:**
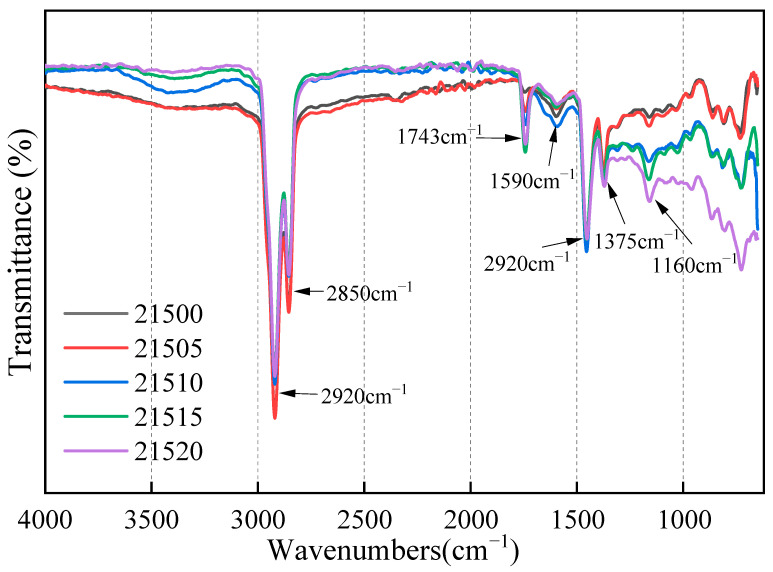
The infrared spectra of B-SCMA with different bio-oil contents.

FTIR experiments were employed to investigate the impact of bio-oil on the chemical composition and functional groups of SCMA at the microscopic level. Figure 13 presents the infrared spectra of UA, SCMA, bio-oil-modified asphalt (BMA), and B-SCMA. Figure 14 displays the infrared spectra of B-SCMA with varying bio-oil contents.

The characteristic peak positions of the modifiers were identified by their C-H stretching vibration, C=O stretching vibration, C=C stretching vibration, etc. The intensity changes of these characteristic peaks reflected the modification effect of the modifiers. As shown in Figure 13, the absorption peaks shared by UA, SCMA, BMA, and B-SCMA in the functional group region are located at 2920 cm^−1^, 2850 cm^−1^, 1590 cm^−1^, 1449 cm^−1^, and 1375 cm^−1^, respectively. The peaks at 2920 cm^−1^ and 2850 cm^−1^ can be attributed to the C-H stretching vibrations of methylene (-CH_2_) and methyl (-CH_3_) groups, while the peak at 1590 cm^−1^ corresponds to the breathing vibration of the benzene ring C=C. The peaks at 1449 cm^−1^ and 1375 cm^−1^ represent the stretching vibrations of methylene and methyl symmetric bonds, indicating the presence of abundant light-weight components and small molecular compounds in these modified asphalts. Compared to the peaks of UA and SCMA, B-SCMA exhibited two new absorption peaks at 1743 cm^−1^ and 1160 cm^−1^, which can be attributed to the stretching vibration of saturated fatty acid esters (C=O) and the stretching vibration of C─O bonds [30,48].

This indicates that the characteristic peaks in B-SCMA can be attributed to bio-oil, SBS/CR, and unmodified asphalt. There were no new functional groups present during the modification process, suggesting that physical blending was synergistic. This also suggests that bio-oil enhances the compatibility between SBS, CR modifiers, and unmodified asphalt in SCMA, facilitating more complete reactions. This implies that bio-oil, when used as a modifier, adjusts and refines the microstructure of SCMA mainly by physical means, rather than by generating new functional groups through chemical reactions.

Figure 14 demonstrates that increasing the bio-oil content did not introduce new characteristic peaks in the modified asphalt. The peak values remain consistent with those in Figure 13, although the intensity of the absorption peaks varies. This finding reaffirms that bio-oil primarily disperses physically in SCMA. Additionally, the saturated aromatic components in bio-oil exhibit low polarity and low molecular weight, creating a low-polarity region around the SCMA structure [49]. The environment further dissolves the low-polarity saturated components and enhances the molecular dispersion within the SCMA, thereby improving its flowability. On a macroscopic level, serial reactions lead to reductions in the viscosity, softening point, and high-temperature properties of modified asphalt. Consequently, a high bio-oil content leads to low viscosity, a decreased softening point, and diminished high-temperature properties.

## 4. Conclusions

To improve the performance of SCMA, castor oil was incorporated as a modifier, and the working mechanism of bio-oil on SCMA under composite CR particle size ratios was analyzed. The results can be summarized as follows:

(1) Conventional tests showed that the addition of bio-oil stabilized the original dense network structure of SCMA by reducing the degradation of the fine CR particles in SCMA and by compensating for the loss of light-weight components in the asphalt. This significantly improved the aging resistance of the SCMA with a composite CR particle size ratio.
(2) The rheological test results revealed that the addition of bio-oil enhances the fatigue resistance and low-temperature cracking resistance of SCMA under a composite CR particle size ratio, particularly at an optimal bio-oil content. Although bio-oil addition negatively impacts the high-temperature properties of SCMA, B-SCMA still outperforms UA in terms of high-temperature properties. Additionally, the addition of bio-oil enhances the temperature sensitivity of SCMA.
(3) The FM and FTIR results indicate that the addition of bio-oil improves the compatibility between asphalt, SBS, and CR, improves the aging resistance of SCMA, and inhibits the degradation of the modifier in asphalt. The distribution of the modifier in bio-oil-treated asphalt is more uniform after aging. However, when the bio-oil content exceeds a certain threshold, it disrupts the stability of the SCMA spatial network structure, weakening the modification effect. Additionally, the modification process of bio-oil, SBS, and CR in asphalt is primarily a physical blending process.
(4) The addition of bio-oil improves the workability of the modified asphalt by promoting an increase in CR recycling while ensuring good mechanical properties. This study provides a theoretical basis for improving the properties of SCMA with bio-oil and investigating its working prospects.
(5) An optimal dosage of bio-oil exists, and we will further analyze the optimal dosage of the modifier and the micro modification mechanism in detail in future work.

## Figures and Tables

**Table 1:** Main technical indicators of the UA.

Properties		Requirements	Result
Penetration (0.1 mm)		80~100	87
Softening point (°C)		>44	47.5
Ductility (5 cm/min, 10 °C)		>30	84
Density (15 °C)		-	1.013
RTFO	Mass change (%)	−0.8~0.8	−0.03
	Penetration ratio (25 °C, %)	>57	60.5
	Ductility (5 cm/min, 15 °C)	>25	31.2

**Table 2:** Main technical specifications of the SBS modifiers.

Properties	Requirements	Result
Appearance	Uniform, granular	Uniform, granular
SBS dosage (%)	≥50	57
Ash content (%)	≤5.0	4.5
Particle mass (g)	≤0.35	0.25

**Table 3:** Crumb rubber technical specifications.

Properties	Requirements	Result
Density (g/cm^3^)	<1.25	1.11
Acetone extract (%)	≤10	7.48
Metal content (%)	≤0.04	0.039
Ash (%)	≤9	8
Carbon black content (%)	≥26	31.5
Rubber hydrocarbon content (%)	≥48	55.5

**Table 4:** Physical properties of bio-oil.

Properties	Unit	Result
Viscosity (60 °C)	Pa·s	76
Density (15 °C)	g/mL	0.97
Flashpoint	°C	245
Moisture content	%	0.25

**Table 5:** The abbreviations used for modified asphalt.

SBS Dosage (%)	CR Dosage (%)	Bio-Oil Dosage (%)	Aging Level	Abbreviation
0%	0%	0%	Original	UA
0%	0%	0%	RTFO	R-UA
0%	0%	0%	PAV	P-UA
2%	15%	0%	Original	21500
2%	15%	0%	RTFO	R-21500
2%	15%	0%	PAV	P-21500
2%	15%	5%	Original	21505
2%	15%	5%	RTFO	R-21505
2%	15%	5%	PAV	P-21505
2%	15%	10%	Original	21510
2%	15%	10%	RTFO	R-21510
2%	15%	10%	PAV	P-21510
2%	15%	15%	Original	21515
2%	15%	15%	RTFO	R-21515
2%	15%	15%	PAV	P-21515
2%	15%	20%	Original	21520
2%	15%	20%	RTFO	R-21520
2%	15%	20%	PAV	P-21520

**Table 6:** Fitting parameters of the rutting index versus temperature.

Asphalt Type	a	b	R^2^
UA	−0.12358	8.01751	0.994
21500	−0.09793	9.21669	0.99983
21505	−0.10117	8.03459	0.99174
21510	−0.1027	8.03637	0.99201
21515	−0.11095	8.39278	0.9959
21520	−0.11517	8.48841	0.99604

## Data Availability

Data are contained within the article.
